# Genome-wide DNA methylation analysis of transient neonatal diabetes type 1 patients with mutations in *ZFP57*

**DOI:** 10.1186/s12881-016-0292-4

**Published:** 2016-04-14

**Authors:** Mads Bak, Susanne E. Boonen, Christina Dahl, Johanne M. D. Hahnemann, Deborah J. D. G. Mackay, Zeynep Tümer, Karen Grønskov, I. Karen Temple, Per Guldberg, Niels Tommerup

**Affiliations:** Wilhelm Johannsen Center for Functional Genome Research, Institute of Cellular and Molecular Medicine, Panum Institute, University of Copenhagen, DK-2200 Copenhagen N, Denmark; Center for Applied Human Molecular Genetics, Kennedy Center, DK-2600 Glostrup, Denmark; Institute of Cancer Biology, Danish Cancer Society, DK-2100 Copenhagen Ø, Denmark; Human Genetics and Genomic Medicine, Faculty of Medicine, University of Southampton, Southampton, SO16 6YD UK; Wessex Clinical Genetics Service, Southampton University Hospitals Trust, Southampton, SO16 5YA UK; Wessex Regional Genetics Laboratory, Salisbury District Hospital, Salisbury NHS Foundation Trust, SP2 8BJ Salisbury, UK; Institute of Cellular and Molecular Medicine, Panum Institute, University of Copenhagen, DK-2200N Copenhagen, Denmark

**Keywords:** Next-generation sequencing, Imprinting disorder, Transient neonatal diabetes, DNA methylation

## Abstract

**Background:**

Transient neonatal diabetes mellitus 1 (TNDM1) is a rare imprinting disorder characterized by intrautering growth retardation and diabetes mellitus usually presenting within the first six weeks of life and resolves by the age of 18 months. However, patients have an increased risk of developing diabetes mellitus type 2 later in life. Transient neonatal diabetes mellitus 1 is caused by overexpression of the maternally imprinted genes *PLAGL1* and *HYMAI* on chromosome 6q24. One of the mechanisms leading to overexpression of the locus is hypomethylation of the maternal allele of *PLAGL1* and *HYMAI*. A subset of patients with maternal hypomethylation at *PLAGL1* have hypomethylation at additional imprinted loci throughout the genome, including *GRB10*, *ZIM2* (*PEG3*), *MEST* (*PEG1*), *KCNQ1OT1* and *NESPAS* (*GNAS-AS1*). About half of the TNDM1 patients carry mutations in *ZFP57*, a transcription factor involved in establishment and maintenance of methylation of imprinted loci. Our objective was to investigate whether additional regions are aberrantly methylated in *ZFP57* mutation carriers.

**Methods:**

Genome-wide DNA methylation analysis was performed on four individuals with homozygous or compound heterozygous *ZFP57* mutations, three relatives with heterozygous ZFP57 mutations and five controls. Methylation status of selected regions showing aberrant methylation in the patients was verified using bisulfite-sequencing.

**Results:**

We found large variability among the patients concerning the number and identity of the differentially methylated regions, but more than 60 regions were aberrantly methylated in two or more patients and a novel region within *PPP1R13L* was found to be hypomethylated in all the patients. The hypomethylated regions in common between the patients are enriched for the ZFP57 DNA binding motif.

**Conclusions:**

We have expanded the epimutational spectrum of TNDM1 associated with *ZFP57* mutations and found one novel region within *PPP1R13L* which is hypomethylated in all TNDM1 patients included in this study. Functional studies of the locus might provide further insight into the etiology of the disease.

**Electronic supplementary material:**

The online version of this article (doi:10.1186/s12881-016-0292-4) contains supplementary material, which is available to authorized users.

## Background

Imprinting is an epigenetic mechanism in mammals by which one allele of certain genes is expressed in a parent-of-origin specific manner [1]. Imprinting is controlled by modifications of sequence elements termed “imprinting control regions” (ICRs). These modifications include DNA methylation as well as acetylation and methylation of histones [2]. Transient neonatal diabetes mellitus 1 (TNDM1) is an imprinting disorder caused by overexpression of the maternally imprinted genes *PLAGL1* and *HYMAI* on chromosome 6q24. There are three major mechanisms related to this disorder: paternal uniparental disomy of chromosome 6, paternal duplications of 6q24 and total loss of maternal methylation at the TNDM1 differentially methylated region (DMR), *PLAGL1*:alt-TSS-DMR, without any detectable DNA sequence changes [3]. Hypomethylation of additional imprinted loci is observed in half of the TNDM1 patients displaying loss of methylation at the TNDM1 locus. In almost half of the TNDM1 patients with multilocus methylation defects (TND-MLMD), homozygous and compound heterozygous mutations were identified in *ZFP57* [4]. Methylation analysis of imprinted loci in these patients have shown that in addition to loss of methylation at *PLAGL1*:alt-TSS-DMR, *PEG3*:TSS-DMR and *GRB10*:alt-TSS-DMR are hypomethylated to different extents, while the *MEST*:alt-TSS-DMR, *KCNQ1OT1*:TSS-DMR and *GNAS-AS1*:TSS-DMR are hypomethylated in some patients [4, 5]. The normal function of ZFP57 is maintenance of imprinting during early embryonic development [6, 7]. Mouse and human ZFP57 binds the methylated hexanucleotide TGCC^me^GC [7, 8] and missense mutations in *ZFP57* can disrupt interaction between the protein and its target sequence [8].

Genome-wide methylation analyses have recently been carried out in patients with TNDM to identify additional aberrantly methylated regions [9–11]. These, analyses were performed using methylation microarrays and were thus restricted to analyzing regions covered by the probes on the arrays. In the present study, we employed a DNA sequencing based approach to detect aberrantly methylated regions in TNDM1 patients with *ZFP57* mutations.

## Methods

### Probands, heterozygotes, and controls

The methylation status at six specific known imprinted loci was previously investigated by methylation specific PCR (MS-PCR) and verified by pyrosequencing [4] in four patients and three heterozygotes selected for this study. The *ZFP57* mutations and methylation status of the patients were:

#### Patient 1

(Proband II-1, family 1): *ZFP57* genotype: c.723C > A / c.723C > A (p.Cys241* / p.Cys241*). Total loss of methylation at *PLAGL1*:alt-TSS-DMR, *GRB10*:alt-TSS-DMR and *PEG3*:TSS-DMR. Partial loss of methylation at *MEST*:alt-TSS-DMR, *KCNQ1OT1*:TSS-DMR, and *GNAS-AS1*:TSS-DMR.

#### Patient 2

(Proband II-2, family 1): *ZFP57* genotype: c.723C > A / c.723C > A (p.Cys241* / p.Cys241*). Total loss of methylation at *PLAGL1*:alt-TSS-DMR, *GRB10*:alt-TSS-DMR and *PEG3*:TSS-DMR. Partial loss of methylation at *MEST*:alt-TSS-DMR and *KCNQ1OT1*:TSS-DMR. Normal methylation at *GNAS-AS1*:TSS-DMR.

#### Patient 3

(Proband II-3, family 2): *ZFP57* genotype: c.257_258delAG / c.257_258delAG (p.E86VfsX28 / p.E86VfsX28). Total loss of methylation at *PLAGL1*:alt-TSS-DMR and *GRB10*:alt-TSS-DMR. Partial loss of methylation at *PEG3*:TSS-DMR. Normal methylation at *MEST*:alt-TSS-DMR, *KCNQ1OT1*:TSS-DMR and *GNAS-AS1*:TSS-DMR.

#### Patient 4

(Proband II-3, family 7): *ZFP57* genotype: c.683G > A / c.838_845delACCCAGGC (p.R228H / p.279fsX1). Total loss of methylation at *PLAGL1*:alt-TSS-DMR and *GRB10*:alt-TSS-DMR. Partial loss of methylation at *PEG3*:TSS-DMR, *MEST*:alt-TSS-DMR, and *GNAS-AS1*:TSS-DMR. Normal methylation at *KCNQ1OT1*:TSS-DMR.

The three heterozygotes had normal methylation at these six loci.

Five unrelated individuals were used as normal controls.

### MBD-seq

Genomic DNA (2 μg) was isolated from peripheral blood leukocytes (PBL) and randomly sheared by nebulization to fragments with an average size of 250 bp. Methylated DNA fragments were isolated using His-tagged methyl binding protein MBD2b using MethylCollector (ActiveMotif) and DNA adaptors were ligated to the ends of the isolated methylated DNA using the NEBNext DNA library preparation kit (New England BioLabs). Ligation products were separated on a 2 % agarose gel and fragments of 200–400 bp in size were isolated from the gel. The isolated fragments were amplified by 15 cycles of PCR using primers binding to the adapter sequences. The resulting library was quantified with picogreen and 36 bases of the fragments were sequenced on a Genome Analyzer IIx (Illumina) following the manufacturer’s protocol.

### Data analysis

Sequences were aligned to the human genome (GRCh37/hg19) using BWA version 0.6.1 [12]. Reads with a mapping quality < 37 and duplicate reads were removed. Methylated genomic regions were identified using MACS version 1.4.2 (*p* < 1e-8) [13]. Methylated regions overlapping more than 50 % were merged. For each methylated region, the number of non-redundant reads was counted for each patient and controls. Regions with less than 20 reads per million reads in all samples were removed. For each patient and *ZFP57*-heterozygous individuals, methylation levels were compared to controls. Aberrantly methylated regions were detected using edgeR [14] (*P* < = 0.05, Benjamini-Hochberg adjusted). Overlap of aberrantly methylated regions with allele-specific methylated regions and mapping relative to Ensembl transcripts were performed using python scripts. The analysis was also performed using BALM [15] as peak caller yielding similar results.

### Bisulfite sequencing

Genomic DNA (1 μg) from PBL was bisulfite converted (Zymo Research). Differentially methylated regions were PCR amplified using 100 ng bisulfite converted DNA as template in a reaction volume of 15 μl (10 μl 10x reaction buffer, 1 μl forward primer (10 μM), 1 μl reverse primer (10 μM), 1 μl dNTPs (2.5 μM each), 0.45 μl MgCl_2_ (50 mM), 0.1 μl Platinum TAQ Polymerase (Invitrogen) and 1 μl DNA template, 8.95 μl H_2_O). Primers for PCR amplification were designed using the Methyl Primer Express Software v1.0 (Appplied Biosystems). Primer sequences were: GDF7_F: AGTTGGGTTATTTGTTGTTAGGA, GDF7_R: ACACRTAAACAACAACAACAAC, MAFF_F: TTGGGGTATTTAAAGGTGTTT, MAFF_R: TACAACTCCTCCTTCTAACACA, GAL3ST1_F: GATAGGGGAAAAAATTAAGGGT, GAL3ST1_R: ACCAACTTAAACCCTACCTCAA, KCNAB3_F: GGGGATATAGGAGTAAGGATTAGG, KCNAB3_R: CTAACAACCCTTAAATCCCAAA, DFNB31_F: TATGGAGTTATTGAAAAAGTTGTGTT, DFNB31_R: ACCCCCTACAAACAACCC, ICAM5_F: AGATGGTTTTAGGTTTGAGGAGT, ICAM5_R: AAACACCCCTACTCCCTACC, PPP1R13L_F: AGAAGGTTTGGGTGTTTTT, PPP1R13L_R: ATCCCTCAATACCCTAACCGTC, ADRA2A_F: TGGTGTGTTGGTTTTTTTTTT, ADRA2A_R: AACTAAACACCCCTCRATACC. PCR conditions were: 96 °C for 10 min., 5 cycles of [95°/15 s; 65°/30s; 72°/30s], 5 cycles of [95°/15 s; 60°/30s; 72°/30s], 30 cycles of [95°/15 s; 55°/30s; 72°/30s] and a final extension at 72 °C for 5 min. For each patient, PCR products were combined and libraries for sequencing on the Genome Analyzer were prepared. Briefly, the combined PCR products were blunt ended and concatemerized by DNA ligation. Ligated DNA was randomly sheared by sonication (Bioruptor, Diagenode), followed by blunt ending and ligation of indexed sequencing adapters. Indexed adapters were designed in a way such that the first 5 bases of each read identify the patient. Adapter oligo 1: 5′-ACACTCTTTCCCTACACGACGCTCTTCCGATCTXXXXXT, adapter oligo 2: 5′-phosphate-xxxxxAGATCGGAAGAGCGGTTCAGCAGGAATGCCGAG, where XXXXX is the index and xxxxx is the complementary bases of the index. The following indexes were used: ACCAAT, AGAGAT, AGTCAT, CAGTCT, CCGGCT, CGATCT, CGTACT, GAGAGT and GCCGGT. Indexed libraries were pooled and sequenced on a Genome Analyzer IIx (Illumina). Reads were aligned to the amplicons using Bismark [16]. For each CpG site, a methylated-unmethylated ratio was calculated. An overall methylated-unmethylated ratio was calculated for each amplicon as the average of all CpG methylation ratios within each amplicon.

## Results and discussion

We used methyl-CpG binding domain protein sequencing (MBD-seq) [17] to generate genome-wide methylation profiles of genomic DNA from peripheral blood leukocytes (PBL) from four TND-MLMD patients with homozygous or compound heterozygous *ZFP57* mutations (*ZFP57*mut/mut), three relatives with heterozygous *ZFP57* mutations (*ZFP57*norm/mut) and five unrelated controls. The four patients have previously been analyzed for changes in methylation levels at selected known imprinted loci [4, 5]. A total of 17,123 methylated regions with at least 20 reads per million reads (RPM) in at least one of the 12 individuals were detected by MBD-seq. More than 20 % (3712 / 17,123) of the methylated regions detected by this method are not covered by probes on the Inifinium HumanMethylation450 Beadchip (Illumina). As patients with imprinting defects can display substantial differences in the number and extent of affected loci [5, 9, 10] they were compared to the controls individually to allow detection of patient-specific methylation changes.

### Validation of method

**Fig. 1:**
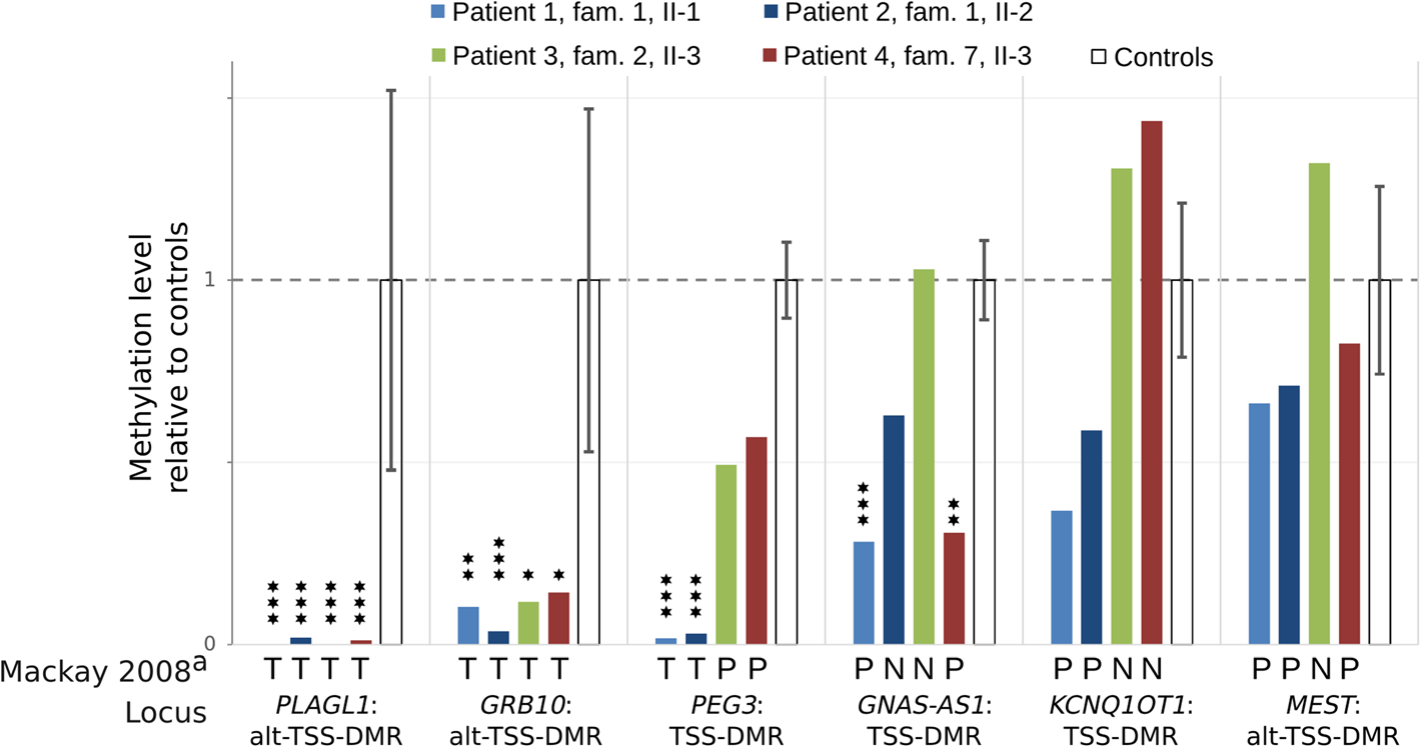
MBD-seq results of previously identified hypomethylated DMRs. Bars show methylation levels relative to controls. Error bars show 1X standard deviation. ^a^: Letters T, P and N below bars indicate methylation results from previous studies [4, 5]. T: Total loss of methylation; P: Partial loss of methylation; N: Normal methylation. Stars indicate level of significance of MBD-seq results: *:P(adjusted) < 0.05; **: P(adjusted) < 0.005; ***: P(adjusted) < 0.0005

**Fig. 2:**
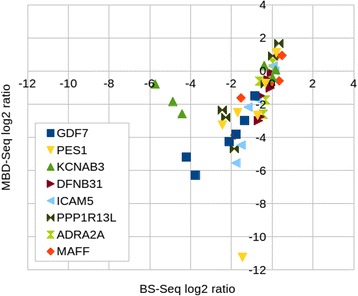
Verification of MBD-seq results by bisulfite sequencing. The plot shows log2 ratios of methylation levels in patients and *ZFP57* heterozygotes versus controls obtained by MBD-seq and BS-seq

To validate the method we compared MBD-seq results with the previous studies of the patients [4, 5]. MBD-seq successfully detected all loci which have previously been shown to be completely hypomethylated (Fig. 1). However, for most of the known partially hypomethylated regions the P-values were above 0.05. To further validate the method, we selected eight hypomethylated regions and verified their methylation status by bisulfite-sequencing (BS-seq) in three patients, three heterozygotes and three controls. The selected regions included regions showing hypomethylation in four (*PPP1R13L*), three (*GDF7*, *DFNB31* and *KCNAB3*), two (*ADRA2A*, *PES1* and *ICAM5*) and one (*MAFF*) of the patients. BS-seq confirmed hypomethylation of these loci and there was good agreement between the changes in methylation levels detected by the two methods (Fig. 2).

### Aberrantly methylated loci

**Fig. 3:**
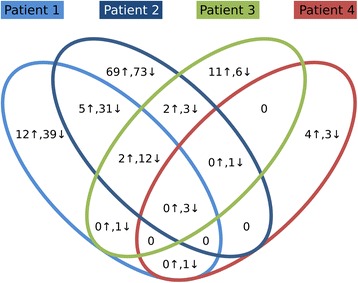
Venn diagram of overlapping aberantly methylated regions in the patients. Up-arrow: hypermethylated regions. Down-arrow: hypomethylated regions. The three hypomethylated regions in common of the four patients are *PLAGL1*:alt-TSS-DMR, *GRB10*:alt-TSS-DMR and *PPP1R13L*

**Fig. 4:**
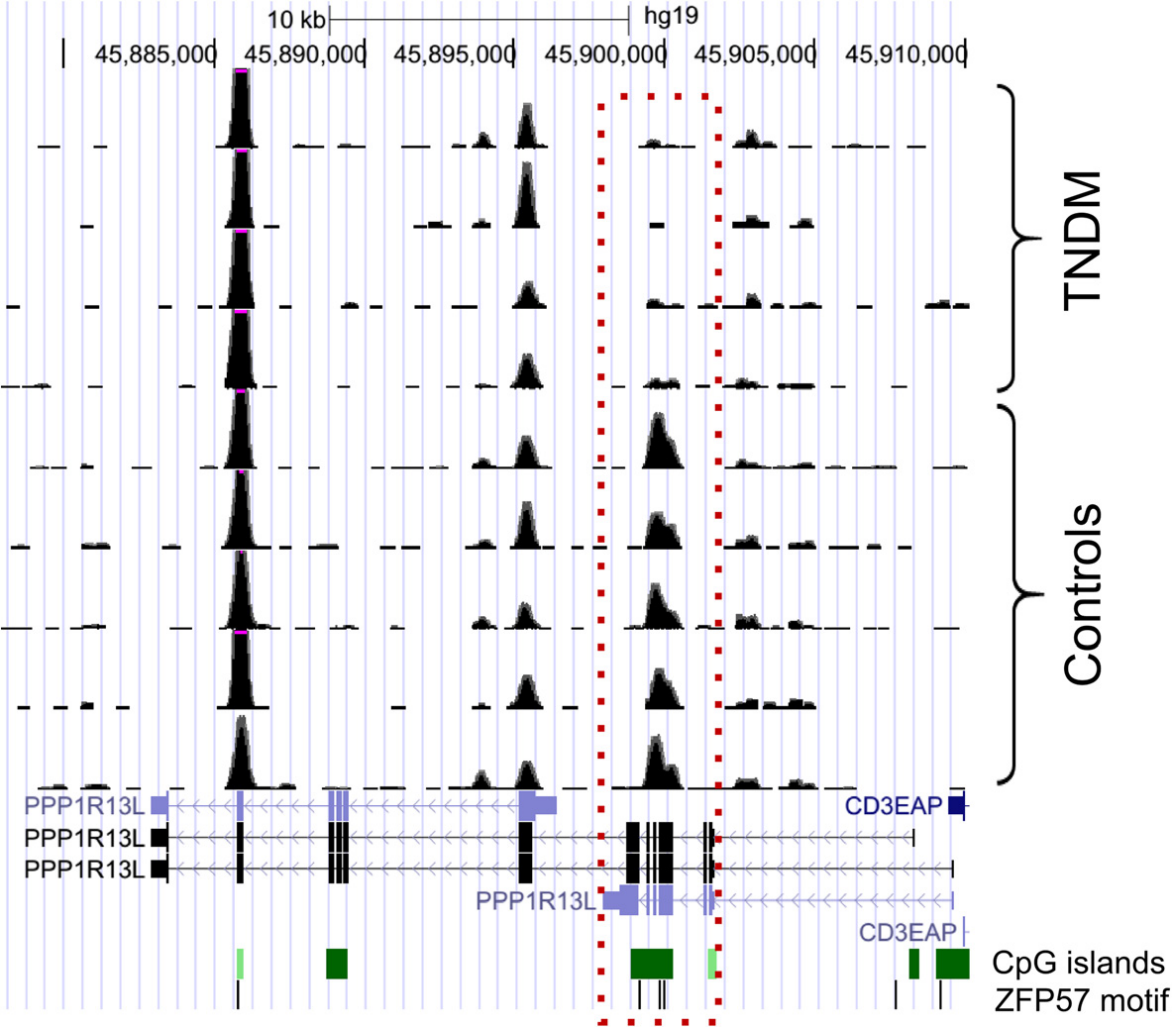
Hypomethylation of *PPP1R13L* in the TNDM1 patients. UCSC Genome Browser screenshot with bedgraph tracks showing MBD-seq reads from the patients (TNDM1) and controls. All patients show hypomethylation of the CpG island indicated by red dots, whereas no changes in methylation is detected elsewhere in the gene. Genomic positions of ZFP57 binding motifs (TGCCGC) are indicated below the CpG islands

We found large variability among the patients regarding the number of affected loci, ranging from 12 regions (8 of these were hypomethylated) to 201 regions (123 of these were hypomethylated) regions in patient 4 and 2, respectively (Additional file 1: Table S1–7 and Fig. 3). In agreement with previous reports, we detected hypomethylation of several imprinted loci, including *PLAGL1*:alt-TSS-DMR [4, 5, 9, 10], *GRB10*:alt-TSS-DMR [4, 5, 9, 10], *PEG3*:TSS-DMR [4, 5, 9], *GNAS-AS1*:TSS-DMR [4, 5, 9, 10], *INPP5F*:Int2-DMR [10] and *NAP1L5*:TSS-DMR [10]. The following known imprinted loci were detected as hypomethylated in the patients: Patient 1: *PLAGL1*:alt-TSS-DMR, *GRB10*:alt-TSS-DMR, *PEG3*:TSS-DMR, *INPP5F*:Int2-DMR, *GNAS-AS1*:TSS-DMR, *NAP1L5*:TSS-DMR and *SLC22A18*; Patient 2: *PLAGL1*:alt-TSS-DMR, *GRB10*:alt-TSS-DMR, *PEG3*:TSS-DMR, *NAP1L5*:TSS-DMR, *SLC22A18*; Patient 3: *PLAGL1*:alt-TSS-DMR, *GRB10*:alt-TSS-DMR; Patient 4: *PLAGL1*:alt-TSS-DMR, *GRB10*:alt-TSS-DMR and *GNAS-AS1*:TSS-DMR. The hypomethylated region within *SLC22A18* is an internal CpG island encompassing exon 4 and 5 of the gene. Methylation changes were not detected at any other known imprinted loci. Docherty and colleagues identified three novel imprinted genes (*NHP2L1*, *WRB* and *PPIEL*) among the hypomethylated regions in TND patients without *ZFP57* mutations [9]. In agreement with the results of the present study, no hypomethylation was observed in patients with *ZFP57* mutations at any of these loci [9]. Three regions were aberrantly methylated - all hypomethylated - in all four patients and two of the them were the known *PLAGL1* and *GRB10* regions. The third region resides within the *PPP1R13L* gene (encoding Inhibitory member of the ASSP family (iASSP) or RelA-associated inhibitor (RAI)) on chromosome 19q13.32. This hypomethylated region is a CpG island encompassing exon 4–6 of *PPP1R13L* (Fig. 4). PPP1R13L is an inhibitory member of the apoptosis stimulating protein of p53 family and overexpression of *PPP1R13L* has been detected in in various cancers [18–21]. In normal tissue, it shows highest expression in the heart, placenta, skin, tounge and prostate (www.biogps.org) [22]. A splice-site mutation in *Ppp1r13l* was identified in Waved3 mice causing open eyelids at birth, wavy coat, and cardiomyopathy [23]. The normal function of gene body CpG islands like the hypomethylated CpG island within *PPP1R13L* is currently not known. They might be involved in regulation of exonic inclusion/exclusion [24] or they could represent “orphan promoters” that are used in early development [25]. The nearest known transcript is a short isoform of *PPP1R13L* (NM_006663) with transcription start site located ~3.5 kb from the hypomethylated CpG island.

In the *ZFP57* heterozygotes, all aberrantly methylated regions were private except for two regions within *ESPNP* and *MUC4* which were hypermethylated in two of the three heterozygotes and a region encompassing *ACTL10*, which was hypomethylated in all heterozygotes. In the patients, *ESPNP* and *ACTL10* were both hypomethylated in three individuals, whereas no changes in MUC4 methylation was detected.

### Aberrantly methylated regions shared between two or more patients

To characterize the differentially methylated common regions, we focused on loci showing methylation changes in two or more patients. A total of 52 loci were hypomethylated and 9 were hypermethylated in at least two patients (Additional file 2: Table S8). Six shared aberrantly methylated regions are not covered by probes on the Inifinium HumanMethylation450 Beadchip (Illumina) (Additional file 2: Table S8). Six known imprinted loci were among the shared hypomethylated regions, including the maternally imprinted *PLAGL1*:alt-TSS-DMR, *GRB10*:alt-TSS-DMR, *PEG3:*TSS-DMR, *GNAS-AS1*:TSS-DMR, *NAP1L5*:TSS-DMR and the maternally expressed *SLC22A18*. The majority of affected regions overlap a CpG island (50 out of 52 hypomethylated and 5 out of 9 hypermethylated). Measured from the center of the regions, 24 hypomethylated and 6 hypermethylated regions map more than 1 kb from the 5′ end of an Ensembl transcript. Thus, more than half of the affected regions are not closely associated with known transcriptional start sites. They are, however, associated with gene bodies. Of those regions not closely associated with transcription start, almost 90 % localizes within Ensembl transcripts and many close to the 3′ end. The normal function of gene body methylation is currently not known, but it is not associated with transcriptional silencing [26].

To analyse whether the identified hypomethylated regions are differentially methylated regions (DMRs) of novel imprinted genes or imprinting control regions (ICRs) regulating parental-of-origin expression of nearby genes, we analysed them for enrichment of regions reported to show allele-specific methylation, enrichment for ZFP57 binding motifs and experimentally investigated a subset of them for allele-specific expression.

We compared the 52 hypomethylated regions with allele-specific methylated regions (AMRs) from MethBase (http://smithlabresearch.org/software/methbase/) [27]. We extracted 92 data sets with AMRs from 22 studies and counted number of datasets showing AMR in the hypomethylated regions. As expected, the known human imprinted DMRs [28, 29] are highly enriched for AMRs. More than 77 % of human imprinted DMRs are detected as AMRs in more than five of the data sets and all germline DMRs (gDMRs) are detected as AMR in more than 15 data sets. Half (51 %) of the hypomethylated regions identified in this study are AMRs in more than 5 data sets whereas only 11 % of all detected methylated regions (1933 / 17,123) was reported as AMRs in more than 5 data sets. However, the five known maternally imprinted loci identified as hypomethylated in this study (*PLAGL1*:alt-TSS-DMR, *GRB10*:alt-TSS-DMR, *PEG3*:TSS-DMR, *GNAS-AS1*:TSS-DMR, *NAP1L5*:TSS-DMR) top the list with more than 36 datasets detecting them as AMRs. Thus, the hypomethylated regions are enriched for AMR. However, allele-specific methylation is not necessarily indicative of imprinting as it is well established that the genotype can influence upon the DNA methylation in *cis* [30–33].

Most of the regions show intermediate methylation in blood cells or other tissues [24] (Additional file 2: Table S8). This was also evident from the BS-seq validation. In the controls the average methylation levels ranged from 25 % to 88 %. The chromatin of the unmethylated allele of many imprinted DMRs are marked by H3K4me2/3 [34]. We downloaded H3K4me2/3 data from blood cells from the ENCODE website (https://www.encodeproject.org/). Of the BS-seq validated regions *ICAM5*, *DFNB31*, *KCNAB3* and *MAFF* showed H3K4me2/3 in most or all cell types whereas *PPP1R13L* and *PES1* did not show the modifications in any of the cell types (Additional file 2: Table S8).

**Fig. 5:**
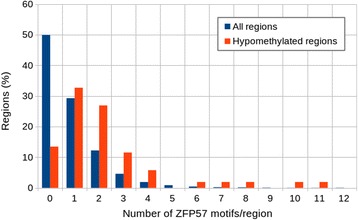
Enrichment of the ZFP57 binding sequence in the hypomethylated regions. The number of occurrences of the TGCCGC sequence were counted in the 52 hypomethylated regions shared between at least two patients (*orange bars*) and in the 17,123 methylated regions detected in any of the samples (*blue bars*)

Mouse and human Zfp57 is involved in maintenance of genomic imprints during early embryogenesis [6]. The protein recognizes the methylated DNA motif TGCCGC (TGCC^me^GC) which is present at all known mouse and some human imprinting control regions [7]. In mice imprinting control regions, the motif is present with an average of two copies [7]. Sequence analysis of the 52 hypomethylated regions in *ZPF57*mut/mut individuals revealed enrichment of the TGCCGC motif compared to all analyzed regions (17,123) (Fig. 5). Approximately 54 % (28/52) of the hypomethylated regions contain two or more ZFP57 binding motif while 21 % (3560 / 17,123) of all methylated regions has two or more copies of this motif. The five hypomethylated regions at the maternally imprinted *PLAGL1*:alt-TSS-DMR, *NAP1L5*:TSS-DMR, *GRB10*:alt-TSS-DMR, *GNAS-AS1*:TSS-DMR *and PEG3*:TSS-DMR have the top five most TGCCGC motifs with 6, 7, 8, 10 and 11 motifs, respectively. Very similar to the hypomethylated regions, 58 % (18/31) of the known human gDMRs [29] contain 2 or more copies of the binding motif.

To further investigate if the hypomethylated regions are imprinted DMRs we genotyped nine trios in the genes adjacent to the eight BS-seq validated hypomethylated regions and sequenced cDNA from peripheral blood samples to investigate for allele-specific expression. We identified informative SNPs in *DFNB31*, *GAL3ST1*, *ICAM5* and *MAFF*, which also were expressed in blood. All showed biallelic expression, suggesting that they are not imprinted. However, we cannot rule out the possibility that they exhibit tissue-specific imprinting.

Thus, the hypomethylated regions in the patients are enriched for allele specific methylated regions and for the ZFP57 binding motif, but not to an extent found at the affected regions that are known imprinted genes. Additionally, none of the regions have been identified as imprinted or candidate imprinted in genome-wide or chromosome-wide screens [35–40], suggesting that they are not associated with imprinted genes. This is in agreement with recent genome-wide studies of patients with multilocus methylation defects other than TND also reporting aberrant methylation at apparently non-imprinted loci [41, 42]. Experiments in mice have shown that ZFP57 in complex with KAP1 and SETDB also binds to methylated non-imprint control regions which looses methylation in *Zfp57* knockout cells [7]. It might be speculated that ZFP57 is also involved in maintenance of methylation at non-imprinted genes in humans.

## Conclusions

In summary, by genome-wide methylation analysis we have expanded the epimutational spectrum of TNDM1 associated with mutations in *ZFP57*. We detected large differences in the number and extent of affected regions in individual patients, but 61 regions were aberrantly methylated in two or more patients. We found one novel region within *PPP1R13L* which was hypomethylated in all TNDM1 patients included in this study. Expansion of the patient cohort will reveal if hypomethylation of this locus is a common feature of TNDM1 and functional studies of the aberrantly methylated regions identified in this study might provide further insight into the etiology of the disease.

### Ethics

Patients were recruited as part of a Danish Imprinting and Methylation study and as part of the UK clinical local research network study: “Imprinting Disorders finding out why”. The Danish study was approved by The National Committee on Health Research Ethics (H-D-2008-079) and Danish Data Protection Agency (2008-41-2565). The UK study was approved by Southampton and South West Hampshire Research Ethics committee (07/H0502/85). Written consent was obtained from patients and guardians for participation in research.

### Consent to publish

Written informed consent was obtained from families for presentation of clinical data and for publication of results. Written consent was given by patients and guardians for the publication of patient and family numbers in anonymised form.

### Availability of data and materials

Data from this study that do not pertain to individual patients are freely available and can be obtained by contacting the corresponding author. We do not have the consent of patients to publish sequencing data in a format required by SRA (http://www.ncbi.nlm.nih.gov/sra), ENA (http://www.ebi.ac.uk/ena) or EGA (https://www.ebi.ac.uk/ega/). Sequencing data will be shared as aligned reads without sequence information. Files with alignments to hg18, hg19 and hg38 are available.

## Additional files

Additional file 1: Tables S1-7. Hypo- and hypermethylated regions detected in patients and heterozygotes. Additional file 2: Table S8. Hypo- and hypermethylated regions detected in at least 2 patients. Additionally, the following characteristics of the regions are included in the table: known imprinting status, CpG island, ZFP57 motifs, microarray probes, bisulphite-sequencing results, allele-specific methylation, intermediate methylation and H3K4me2/me3.
